# Urachal yolk sac tumor penetrating the bladder as a diagnostic challenge: a case report and review of the literature

**DOI:** 10.1186/s13000-022-01190-y

**Published:** 2022-01-14

**Authors:** Vladimír Šámal, Tomáš Jirásek, Vít Paldus, Igor Richter, Ondřej Hes

**Affiliations:** 1 Department of Urology, Krajská Nemocnice Liberec a.s, Liberec, Czech Republic; 2 Department Of Urology, Faculty of Medicine in Hradec Králové, Charles University, Prague, Czech Republic; 3 Department of Pathology, Krajská Nemocnice Liberec, a.s, Liberec, Czech Republic; 4 Department of Oncology, Krajská Nemocnice Liberec, a.s, Liberec, Czech Republic; 5 Department of Oncology, First Faculty of Medicine, Charles University and Thomayer Hospital, Prague, Czech Republic; 6 Charles University and University Hospital Pilsen, Pilsen, Czech Republic

**Keywords:** Extragonadal yolk sac tumor, Urachal yolk sac tumor, Yolk sac tumor

## Abstract

**Background:**

Yolk sac tumor (YST) is a germ cell tumor. It is primarily located in the gonads but can also occur extragonadally (extragonadal yolk sac tumor - EGYST), most commonly in the pelvis, retroperitoneum or mediastinum. Only a few YSTs of the urachus have been described.

**Case report:**

We present a rare case report of a 37-year-old male with episodes of macroscopic hematuria. The histological specimen obtained by transurethral resection showed a solid, and in some parts papillary infiltrative, high-grade tumor with numerous areas of marked nuclear atypia and clear invasion between the detrusor bundles. Glandular pattern has been observed in only minority of the tumor. Immunohistochemistry showed significant positivity for GPC3, SALL4 and cytokeratins AE1/AE3, while KRT7 and GATA3 were negative. We concluded that the biopsy findings were consistent with urothelial carcinoma with infrequent YST differentiation. In definitive surgical specimens we found a malignant epithelial, glandular and cystically arranged tumor of germinal appearance arising from urachus. The surrounding urothelium was free of invasive or in situ tumor changes. We reclassified the tumor as a urachal YST.

**Conclusion:**

EGYST was suspected because glandular and hepatoid structures were found, but the presence of these structures should be verified by immunohistochemistry.

## Introduction

Yolk sac tumor (YST) is a type of germ cell tumor that originates predominantly in the gonads. However, it can also occur extragonadally (extragonadal yolk sac tumor - EGYST). Extragonadal forms represent 1–5% of germ cell tumors and are considered to be a consequence of the incorrect migration of germ cells during embryogenesis [1]. The incidence of EGYST is 1.8–3.4/1 million people, affecting males and Caucasians more often [2]. The most common extragonadal locations of YST include the pelvis, retroperitoneum, and mediastinum [3–5]. In a number of somatic tumors, YST differentiation can be observed in some parts of the tumor, and somatic derivation of YST has been considered. The most common tumors with YST differentiation include adenocarcinomas, immature teratomas and serous carcinomas in the pelvic, sacrococcygeal and gastrointestinal regions [4, 6].

The urachus is a remnant of the embryonic structure connecting the bladder to the umbilicus that is present in adulthood. Tumors of the urachus are rare, and the most common tumor type is adenocarcinoma. Only a few cases of primary YST of the urachus have been described [7–10].

This case report presents the diagnostic challenge of EGYST penetrating into the bladder.

## Case report

**Fig. 1:**
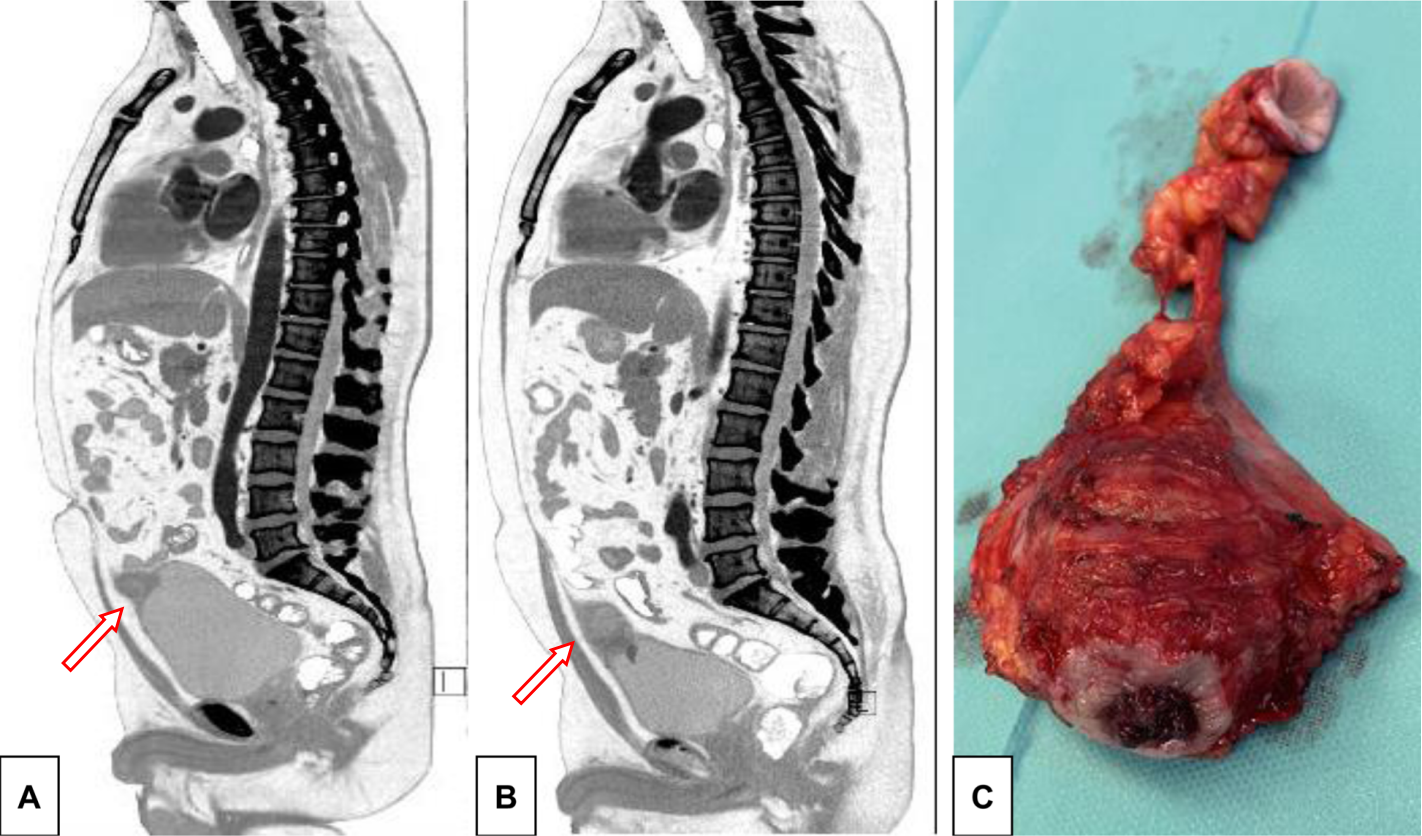
CT scan and surgical specimen. **A** At the time of diagnosis, a CT scan showed a 25x25x20 mm tumor in the vertex of the bladder, which was mostly located extravesically. No pathologically enlarged lymph nodes or distant metastases were demonstrated. **B** After six cycles of chemotherapy, the size of the tumor increased, and its base extended widely into the bladder. The tumor was 45x50x40 mm in size, and the CT image showed its invasion into the urachus. **C** A surgical specimen of the resected tumor with urachus and umbilical tissue is shown. There was evident invasion into the urinary bladder

A 37-year-old man, who was otherwise healthy, suffered episodes of macroscopic hematuria. A solid polypoid tumor of 30 mm in diameter was evident in the bladder dome during the cystoscopy examination. A CT scan showed an exophytic tumor in the bladder dome infiltrating the perivesical fat (Fig. 1). We performed diagnostic transurethral resection. Macroscopically, the tumor was mostly solid and rigid on resection with small areas of necrosis. The tumor did not have the macroscopic appearance typical of urothelial carcinoma.

Histologically, the specimen contained multiple, partially thermally bruised areas, most of which consisted of solid and in places papillary infiltrative tumor components. The tumor contained numerous areas with prominent nuclear atypia and distinct invasion into the subepithelial connective tissue and between the trapped bundles of detrusor smooth muscle. The tumor showed necrotic foci and some of the tumor cells showed clear cytoplasm, with conspicuous eosinophilic globules in places. A glandular tumor pattern was present in up to 5% of areas. Based on the microscopic appearance on HE sections we concidered so-called variant histology within the spectrum of urothelial carcinoma before receiving the immunohistochemistry results.

**Fig. 2:**
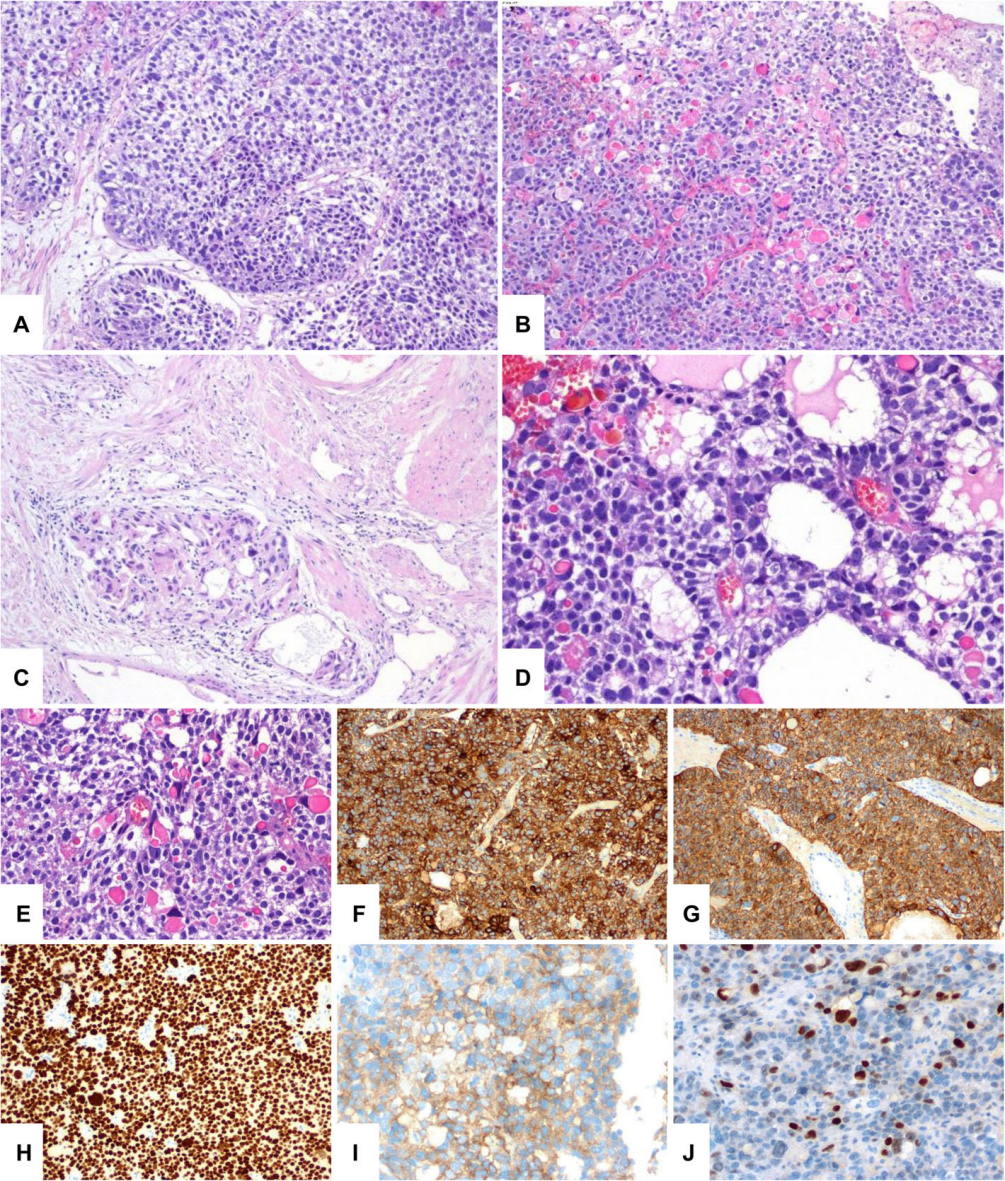
**A** Superficial areas of the resected tumor showed urothelial carcinoma with papillary morphology. **B** Different parts of similar tumors are shown, including solid and adenoid areas with clear cells and hyaline globules (stars). **C** Carcinoma nests were found among smooth muscle fascicles of the bladder wall. **D** There were glandular formations within the tumor. **E** There were clear cells and hyaline globules. Immunostaining: (**F)** Glypican-3 was present in tumor cells. **G** Keratins AE1/AE3 were present in tumor cells. **H** SALL4 was present in tumor cell nuclei. **I** AFP was present in tumor cells. **J** CDX2 was present in tumor cell nuclei

Immunohistochemically, the tumor expressed glypican-3 (GPC3), SALL4, broad-spectrum keratins (AE1/AE3) and alpha-fetoprotein (AFP). The tumor showed localized expression of synaptophysin and CDX2 (Fig. 2). The tumor was negative for epithelial membrane antigen (EMA), HepPar1, vimentin, keratin7, placental-like alkaline phosphatase (PLAP), CD117, CD30, OCT3/4, hCG, p63, and GATA-3.

The serum AFP level was 446 kU/L (normal range 0.7–7.3 kU/L), and the hCG and LD levels were within the normal ranges. The sonography findings on the testes showed no tumor changes or calcifications. Fluorescence in situ hybridization (FISH) using locus-specific probes for NTF3 (12p13) and STAT6 (12q13) (SureFISH Agilent Technologies, Santa Clara, CA, USA) showed no gains in chromosome 12p. The presence of isochromosome was not tested.

Due to atypical changes suggestive of diagnosis of urothelial carcinoma and foci of YST differentiation the tumor was histologically classified as high-grade urothelial carcinoma with YST differentiation.

The patient refused surgery and was treated with etoposide/cisplatin (EP) chemotherapy. After 2 cycles of EP, the AFP level rose to 2294 kU/L. The chemotherapy regimen was changed to the paclitaxel/ifosfamide/cisplatin (TIP) salvage regimen. After 4 cycles of TIP, the tumor size increased according to control CT. The AFP level plateaued at 2034 kU/L. Control cystoscopy showed an exulcerated lesion in the bladder dome bordered by a minimal circumscribed rim of reddened mucosa. Ultimately, resection of the bladder dome, urachus and umbilicus was performed 6 months after the diagnosis (Fig. 1).

**Fig. 3:**
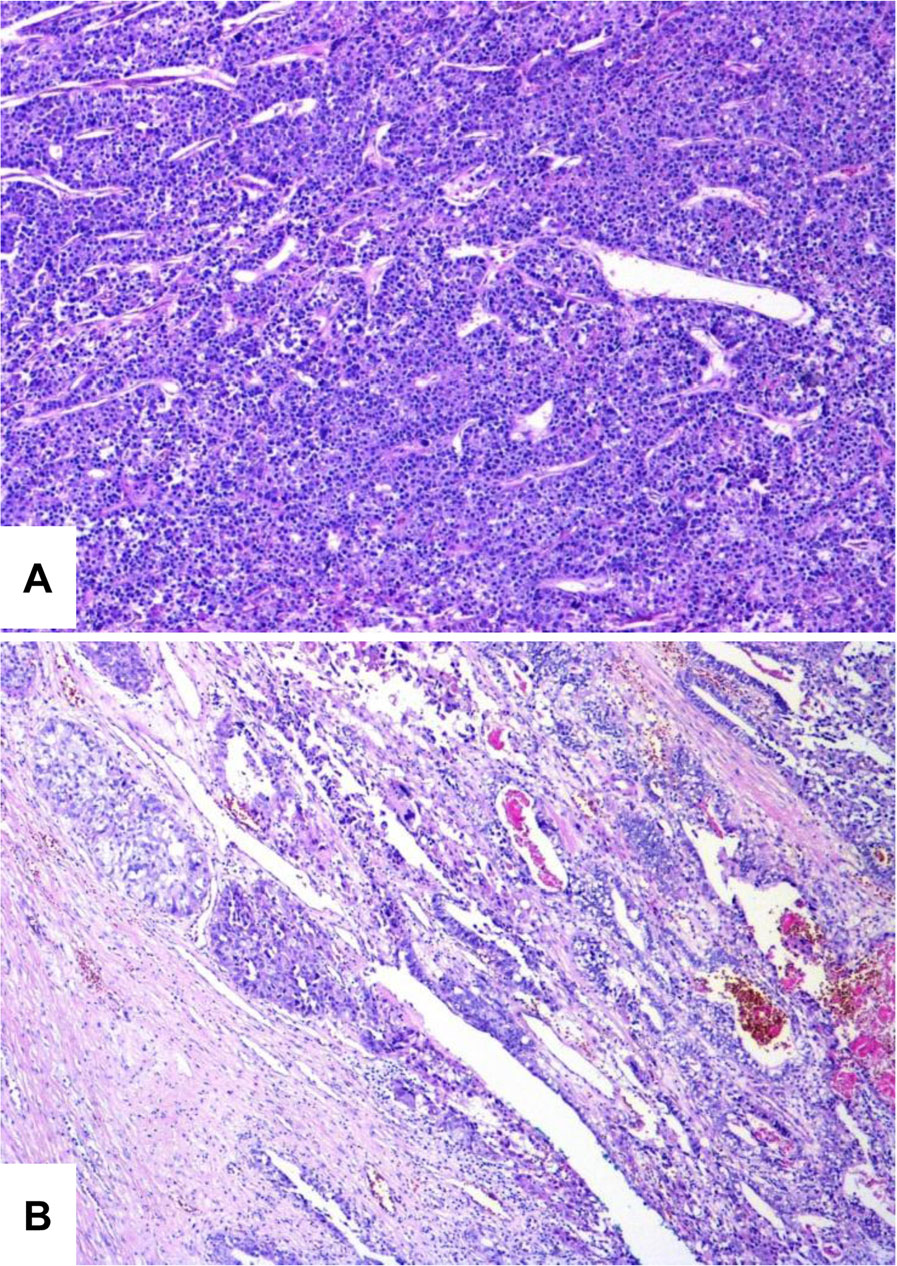
**A** Histological findings from the resected tumor specimen showed a solid trabecular, sporadically glandular and cystically arranged malignant tumor of germinal appearance. **B** The transition to urothelium was abrupt, without any dysplasia or in situ change of the urothelium

Tumor was localized in the distal part of the urachus and penetrated the bladder dome. A malignant epithelial tumor with a pattern typical of YST, mainly glandular and with a cystic arrangement, was noted. Satellite foci of the same tumor were observed outside the main tumor mass. The transition from the urothelium to the YST in an excised part of the bladder was very abrupt, without any dysplasia or in situ changes in the urothelium (Fig. 3). We observed positive staining for SALL4 and GPC3. The tumor showed positive staining for keratins (AE1/AE3), and the intensity of the staining reaction was mostly strong within the cytoplasm of neoplastic cells; however, small foci of cells with weaker staining positivity were also detected within the tumor mass.

Immunostaining for GATA3, OCT3/4, KRT 7, chromogranin A and synaptophysins was negative. FISH did not detect any gain in the 12p region.

Based on these findings, we reclassified the tumors as EGYST penetrating into the bladder. Six months after treatment, the patient had no evidence of disease recurrence on CT and serum AFP levels were within the normal range.

## Discussion

The initial histology findings obtained from transurethral resection showed both solid and papillary tumor components, invasion between the detrusor bundles and a focal glandular component that showed GPC3, SALL4, and AFP positivity. The presence of papillary tumor changes imitating urothelial tumor and YST structures led us to a working diagnosis of urothelial carcinoma with YST differentiation. The definitive histological findings obtained after surgery caused us to correct the diagnosis to EGYST, and the tissue surrounding the urothelium and the urothelium itself were free of tumor changes. Atypical changes suggestive of the diagnosis of urothelial carcinoma were later interpreted as reactive.

The histological variability of YST and its ability to mimic somatic tumors may cause diagnostic difficulties [11]. Compared with other germ cell tumors, YST presents very varied and diverse histological findings. In particular, its occurrence in extragonadal locations and outside the typical age range cause diagnostic challenges. YST can occur anywhere along the midline. From a urogenital pathology perspective, involvement of the renal pelvis [6], prostate [12], seminal vesicle [13], bladder [14, 15], and retroperitoneum [5] is known. YSTs are highly aggressive tumors that can metastasize lymphatically and hematogenously. EGYSTs occur at various ages with a tendency for a worse prognosis with increasing age. Typically, EGYSTs manifest in childhood and in young women [11]. The patient presented in our case report was 37 years of age. Only one case of urachal YST reported thus far involved a patient aged 44 years [9]. In the rest of the reported cases, the patients were children under 2 years of age [7, 10].

YST differentiation of somatic tumors is relatively rare, and reported cases of YST differentiation of urothelial tumors are even less common [6, 16, 17]. Histologically, tumors can contain both YST and somatic tumor structures. The YST component corresponds to the germ cell tumor, and it is not uncommon for the YST component to grow larger than the somatic tumor component due to its higher proliferative activity [18]. Somatic malignancies with YST differentiation are characterized by the presence of different patterns, the most typical being the glandular pattern [1]. In our case, solid and papillary patterns were the main patterns found in the biopsy. The glandular pattern that is usually described as typical for YST differentiation was observed only focally (Fig. 2). There are available data on AFP-producing urothelial tumors [19–21]. Most of these tumors present hepatoid tissue areas in the context of adenocarcinoma or urothelial carcinoma and show AFP positivity. In our case, no area with a hepatoid pattern similar to that in the YST were observed. Samaratunga et al. published a case report of urothelial carcinoma of the renal pelvis with focal hepatoid adenocarcinoma differentiation, and the tumor showed strong AFP immunoreactivity and serum AFP positivity [16]. There are also documented cases of bladder adenocarcinomas that have shown both immunoreactivity for AFP and elevated serum AFP levels [16]. Recently, a case of urothelial tumor with YST differentiation was presented by Espejo-Herrera et al. The patient was a 76-year-old male with a history of recurrent urothelial carcinoma of the bladder [17].

In addition to the abovementioned microscopic features, immunohistochemical methods are essential for accurate diagnosis. Extragonadal tumors usually show the same immunoreactivity as their gonadal counterparts [11]. AFP and GPC3 are characteristic immunohistochemical markers of YST, which may correlate with their serum levels [1, 22]. Some stem and pluripotent cell antigens (SALL4, Lin28 or IMP3) may also be helpful in diagnosis.

AFP remains the standard marker for YST, even though it can also be produced by several non-germ cell tumors, especially those of the female genital tract, and tumors of endodermal origin with a frequent hepatoid component [1]. AFP staining shows strong granular cytoplasmic positivity, and AFP may also be expressed in hyaline globules, although this is not always present [1]. Determination of serum AFP is useful in the diagnosis and follow-up of patients with YST or EGYST.

Most YSTs show positivity for AFP, GPC3 and SALL4 [23–25]. In contrast, most differentiated epithelial markers, such as EMA and KRT17 shows negative reactions. Inconsistent results were shown for the markers HepPar1, which may be positive in hepatoid pattern regions, and CDX2, which may be positive in glandular pattern regions of the tumor. There has also been a report of pure urothelial carcinoma with SALL4 positivity [26]. Most published case reports of urachal YSTs, including our case report, reported positivity for at least AFP and keratins [8, 9].

Genetic alterations are common in germ cell tumors, including isochromosome 12p, p53 alteration, and other changes [27, 28]. We did not find a gain of 12p when we tested for numerical changes in the 12p region by FISH. Extragonadal germ cell tumors do not appear to share a common genetic basis with their gonadal counterparts.

Prognostically, extragonadal forms of germ cell tumors are significantly worse than those in the gonads [29]. Tumors with a YST component are diagnosed at a younger age and may be more likely to be pure EGYSTs [6]. The determination of whether a tumor is EGYST or a somatic tumor with YST differentiation also has a major impact on therapy. While most gonadal YSTs respond relatively well to chemotherapy, a benefit of chemotherapy for EGYST is more likely to be seen in younger patients. The benefit of chemotherapy for somatic tumors with YST differentiation is unclear [6]. In the case of advanced or metastatic germ cell cancer, cisplatin-based chemotherapy is the mainstay. Five-year survival rates range from 40 to 90%, with a more favorable prognosis for seminomas or retroperitoneal tumors than for nonseminomas or mediastinal tumors [29].

This case includes a rare presentation of urachal EGYST. Chemotherapy was not associated with the achievement of a curative response. Remission was achieved only after radical surgery. The finding of glandular and hepatoid structures in the tumor is suspicious for EGYST. Serum AFP marker positivity may be an advantage. Suspicion of EGYST should be verified by immunohistochemistry.

## Data Availability

All generated or analyzed during this study are included in this article.

## Abbreviations

YST: Yolk sac tumor
AFP: Alpha fetoprotein
GPC3: Glypican 3
SALL4: Sal-like protein 4
AE1/AE3: Keratin AE1/AE3
CDX2: Caudal type homeobox 2
EMA: Epithelial membrane Antigen
HepPar 1: Hepatocyte Paraffin 1
OCT3/4: Octamer binding transcription factor 3/4
KRT7: Keratin7
PLAP: Placental alkaline phosphatase
CD117: Monoclonal anti-human CD117
CD30: Monoclonal mouse anti-human CD30
hCG: Human chorionic gonadotropin
p63: Transformation-related protein 63.
GATA3: GATA binding protein 3
12p: Short arm of chromosome 12
NTF3: Neurotrophin 3
STAT6: Signal transducer and activator of transcription 6
